# Structure and function of STAC proteins: Calcium channel modulators and critical components of muscle excitation–contraction coupling

**DOI:** 10.1016/j.jbc.2021.100874

**Published:** 2021-06-12

**Authors:** Britany Rufenach, Filip Van Petegem

**Affiliations:** Department of Biochemistry and Molecular Biology, Life Sciences Institute, University of British Columbia, Vancouver, Canada

**Keywords:** adaptor protein, allosteric regulation, calcium channel, excitation–contraction coupling, structure–function, ryanodine receptor, skeletal muscle, CaM, calmodulin, CDI, calcium-dependent inactivation, CTD, C-terminal domain, EC, excitation-contraction, MH, malignant hyperthermia, NAM, Native American myopathy, RyR1, ryanodine receptor 1, SR, sarcoplasmic reticulum, STAC, Src homology three and cysteine rich domain, VDI, voltage-dependent inactivation, VSD, voltage-sensing domain

## Abstract

In skeletal muscle tissue, an intriguing mechanical coupling exists between two ion channels from different membranes: the L-type voltage-gated calcium channel (Ca_V_1.1), located in the plasma membrane, and ryanodine receptor 1 (RyR1) located in the sarcoplasmic reticulum membrane. Excitable cells rely on Ca_v_s to initiate Ca^2+^ entry in response to action potentials. RyRs can amplify this signal by releasing Ca^2+^ from internal stores. Although this process can be mediated through Ca^2+^ as a messenger, an overwhelming amount of evidence suggests that RyR1 has recruited Ca_V_1.1 directly as its voltage sensor. The exact mechanisms that underlie this coupling have been enigmatic, but a recent wave of reports have illuminated the coupling protein STAC3 as a critical player. Without STAC3, the mechanical coupling between Ca_v_1.1 and RyR1 is lost, and muscles fail to contract. Various sequence variants of this protein have been linked to congenital myopathy. Other STAC isoforms are expressed in the brain and may serve as regulators of L-type Ca_V_s. Despite the short length of STACs, several points of contacts have been proposed between them and Ca_V_s. However, it is currently unclear whether STAC3 also forms direct interactions with RyR1, and whether this modulates RyR1 function. In this review, we discuss the 3D architecture of STAC proteins, the biochemical evidence for their interactions, the relevance of these connections for functional modulation, and their involvement in myopathy.

Excitation–contraction coupling in muscle is the process whereby an electrical signal—a depolarization of the plasma membrane—leads to muscle contraction. The main step in this process is the release of Ca^2+^ from the sarcoplasmic reticulum (SR) in response to the depolarization. This process requires a highly specialized membrane system. The muscle plasma membrane, the sarcolemma, contains invaginations known as transverse tubules (T-tubules), which are flanked on either side by parts of the SR, forming the “triad” (Fig. 1). This allows for a very specialized signaling that involves two membrane proteins. Voltage-gated calcium channels (Ca_V_s) located in the T-tubular membrane undergo conformational changes upon depolarization, resulting in the influx of Ca^2+^ from the extracellular space. Ryanodine receptors (RyRs), located in the SR membrane proximal to the Ca_V_s, are then triggered to open, resulting in the release of Ca^2+^ from the SR. RyRs thus amplify the Ca^2+^ signal, a process that is key for muscle contraction. This process is fundamentally different in cardiac *versus* skeletal muscle. In both tissues, Ca^2+^ ions that enter through Ca_V_s can bind to sites on the nearby RyRs, stimulating their opening. Ca^2+^ release by one RyR can also trigger neighboring RyRs. This process is known as calcium-induced calcium release (1). In skeletal muscle, however, the influx of Ca^2+^ is not a prerequisite for opening of RyRs (2, 3, 4). A widely accepted model is that conformational changes in Ca_V_1.1, the skeletal muscle isoform, are transmitted mechanically to ryanodine receptor 1 (RyR1). Thus, RyR1 has recruited Ca_V_1.1 as its voltage sensor, allowing for voltage-induced calcium release. This coupling is not unidirectional, as the presence of RyR1 also affects the gating of Ca_V_1.1, including an increased current amplitude and altered activation kinetics (5, 6). Also called “retrograde” coupling, RyR1 seems to behave as an auxiliary protein that modulates Ca_V_1.1.

**Figure 1:**
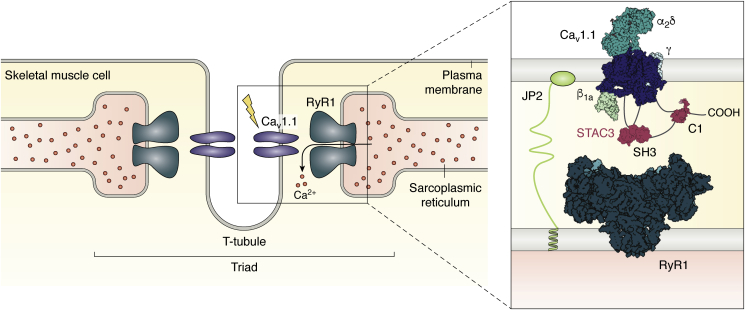
**Overview of the specialized skeletal muscle membrane systems.** Cav1.1 resides in the T-tubule, an infolding of the plasma membrane. The T-tubule is flanked by parts of the SR, in which RyR1 is situated. The overall configuration of SR–plasma membrane–SR constitutes the triad. For simplicity, the figure shows one Cav interacting with one RyR; in reality, four Cav proteins interact with one RyR homotetramer. The inset shows a more detailed view of selected triad proteins and the proposed interactions of STAC proteins. The SH3 domains of STAC3 bind to the cytoplasmic II-III loop of Ca_v_1.1, while the C1 domain and/or the U-motif are postulated to interact with the C-terminal domain. Conclusive evidence of whether STAC3 also interacts with RyR1 remains to be found. Ca_v_1.1, RyR1, STAC3, β_1a_, and Junctophilin2 (JP2) constitute the core components of the skeletal muscle EC coupling machinery. RyR1, ryanodine receptor 1; SR, sarcoplasmic reticulum.

Coupling between Ca_v_s and RyRs is not exclusive to muscle tissue. For example, it has also been observed in the central nervous system, including cerebellar granule cells, sensory neurons, and hippocampal neurons (7, 8, 9, 10). In some cell types coupling may occur *via* direct interactions (11). Such signaling may regulate neuronal gene expression and synaptic plasticity (12).

Because of the critical role of these channels in muscle function, any mutation that affects either protein can have devastating consequences, often resulting in congenital myopathies. Similarly, mutations in the coupling protein STAC3 have been linked to “Native American myopathy” (a.k.a. “STAC3 disorder”), a neuromuscular disease that shares symptoms usually ascribed to RyR1 mutations. Thus, a complete picture of both the normal and pathophysiological mechanisms requires a full understanding of the entire “excitation–contraction complex.” To sketch a more precise role for STAC3 in this process, we first highlight the main features of two major players in excitation-contraction (EC) coupling: the channels Ca_V_1.1 and RyR1.

## Voltage-gated calcium channel architecture

**Figure 2:**
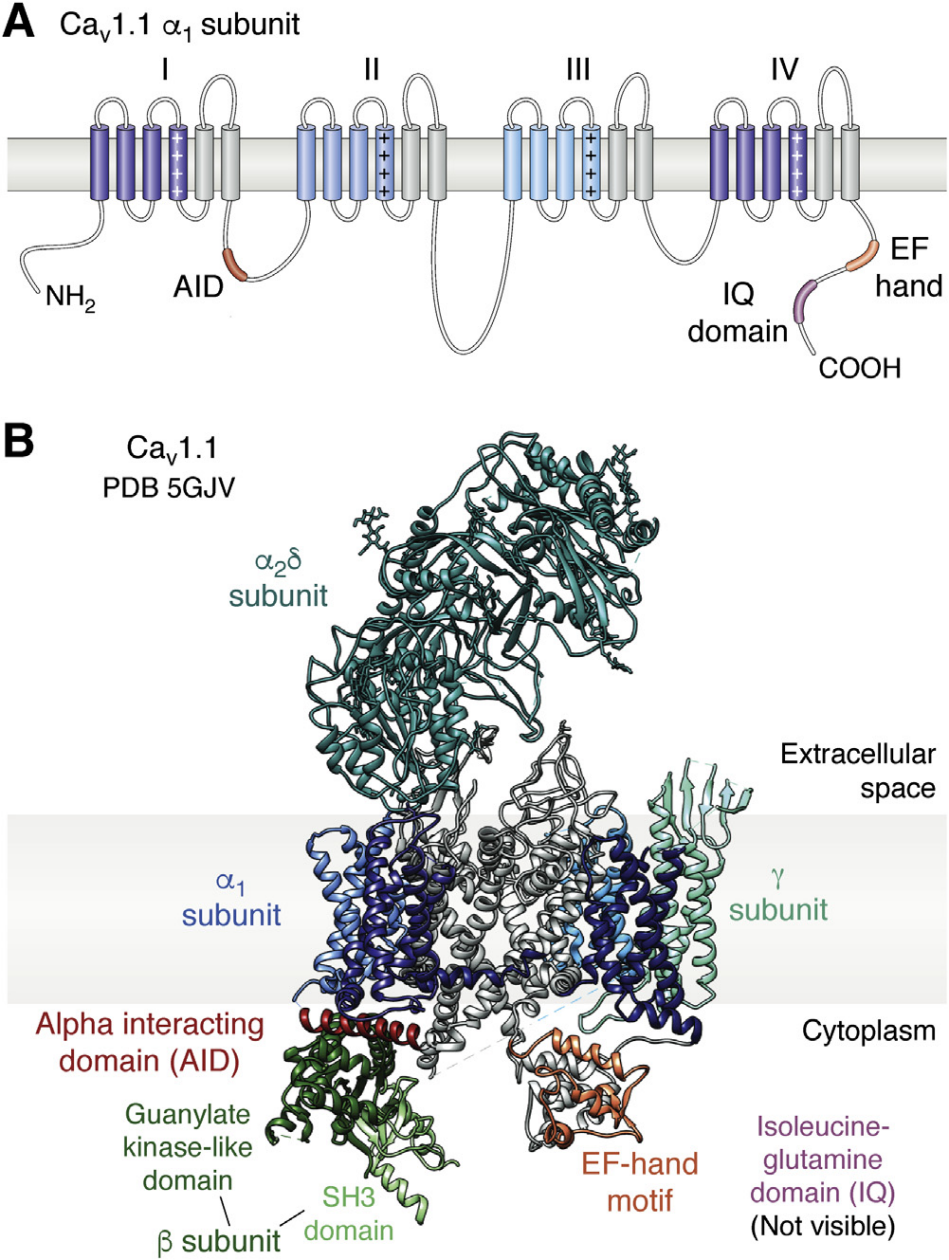
**Structure of the skeletal muscle voltage-gated calcium channel, Ca**_**v**_**1.1 (Protein Data Bank ID:****5GJV****).***A*, domain architecture of the α1 subunit. Each homologous repeat comprises a voltage sensing (*shades of blue*) and pore-forming (*gray*) portion. The repeats are connected *via* mostly flexible linkers. *B*, *ribbon* representation of Ca_v_1.1 with the subunits labeled. Each voltage-sensing domain in the α_1_ subunit is colored a shade of *blue*, corresponding to the colors in (*A*). The beta subunit comprises two domains: the guanylate kinase-like domain (*dark green*) and the SH3 domain (*light green*). AID, alpha interacting domain; EF, EF-hand motif; IQ, isoleucine–glutamine domain (not visible in any cryo-EM structure of Ca_V_1.1).

Voltage-gated calcium channels are plasma membrane channels that are key regulators of many physiological processes in excitable cells (13). Upon depolarization of the plasma membrane, these channels open, resulting in the influx of extracellular Ca^2+^ into the cytosol. All Ca_V_s contain an α_1_ subunit that is responsible for voltage sensing and includes the permeation pathway for Ca^2+^. Ten isoforms of α_1_ subunits are known in humans, which can be grouped into three classes: Ca_v_1 (L-type), Ca_v_2 (P/Q-, R-, N-type), and Ca_v_3 (T-type). The α_1_ subunit conforms to the classic voltage-gated calcium and sodium channel architecture, consisting of four homologous but nonidentical repeats (Fig. 2). Each repeat contains six transmembrane (TM) helices (S1–S6): S1–S4 form the voltage-sensing domain (VSD), whereas S5 and S6 contribute to the pore-forming domain. These repeats are connected by variable, disordered intracellular loops (I-II loop, II-III loop, III-IV loop). The N and C termini are also cytoplasmic with the latter hosting interactions with several regulatory proteins (14). Members of the Ca_V_1 and Ca_V_2 families also contain auxiliary subunits, including an intracellular β subunit, an extracellular α_2_δ subunit and, in some cases, a 4-TM γ subunit.

Skeletal muscle exclusively expresses Ca_v_1.1, whereas cardiac myocytes mostly express Ca_V_1.2 with a small amount of Ca_V_1.3 in atrial myocytes (13). Cryo-EM studies have shed light on the structure of Ca_V_1.1, obtained from rabbit skeletal muscle tissue (Fig. 2) (15). The Ca_V_β subunit associates with the I-II loop and mediates trafficking and the activation and inactivation kinetics of the channel. Although poorly resolved in the cryo-EM density, crystal structures have revealed its architecture in more detail, resolving two structured domains (an SH3 and Guanylate Kinase or GK domain). The GK domain forms high-affinity interaction with the I-II loop (16, 17, 18, 19, 20). The two domains are separated and flanked by disordered regions. The α_2_δ subunit is entirely extracellular and thought to be membrane anchored through a lipid modification (21). The γ subunit is mostly membrane embedded, forming interactions with the VSD of Repeat IV.

In the context of EC coupling, cytosolic regions and subunits are possible candidates for mediating mechanical coupling. Indeed, a main role is attributed to the loop connecting repeats II and III (II-III loop) in Ca_V_1.1, containing essential elements for the coupling to occur (22, 23). In addition, the Ca_V_β1_a_ subunit, a particular splice variant of the β1 isoform, is also indispensable and its disordered C-terminal tail was found to be essential (24, 25, 26, 27). However, to date, unambiguous proof of interactions of these elements with RyR1 is lacking.

## Ryanodine receptor architecture

RyRs are the largest known ion channels, with molecular weights exceeding 2 MDa (28). Three isoforms (RyR1–3) exist in humans and all mammalian species. Skeletal muscle mostly expresses RyR1, with a small amount of RyR3. RyR2 is the predominant isoform in cardiac myocytes. However, RyRs are found throughout the body, including the central nervous system (13, 29). All RyRs form homotetrameric assemblies, with each subunit containing ~5000 amino acid residues.

**Figure 3:**
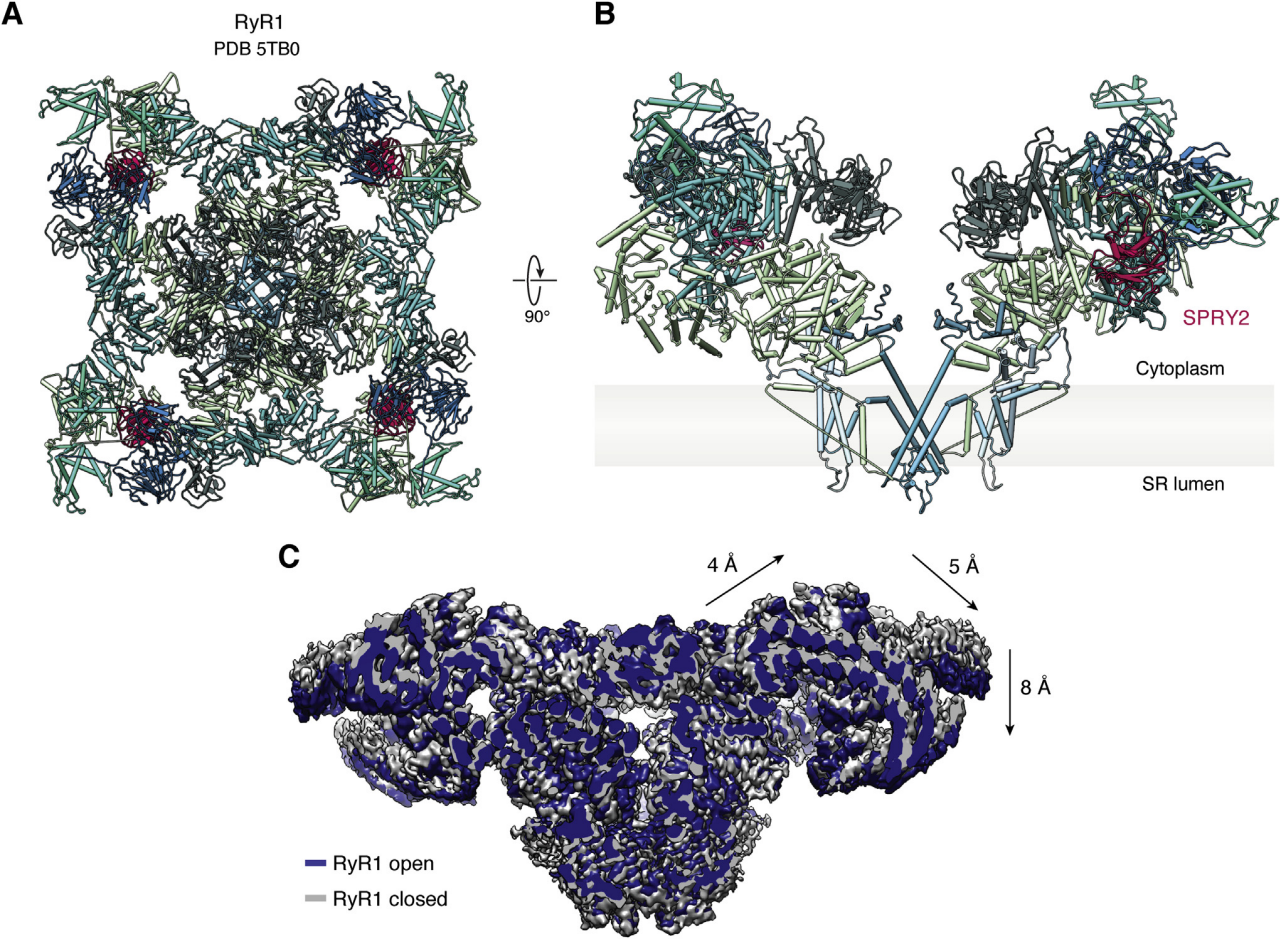
**Structures of RyR1.***A*, *top view* of rabbit RyR1, looking toward the SR membrane (Protein Data Bank ID: 5TB0). *B*, *side view*, from within the plane of the SR membrane, of two opposing subunits of RyR1. The SPRY2 domain is highlighted in *pink*. Although solvent accessible, it is not at the *top surface* exposed on the cytosolic face. *C*, the allosteric movements of RyR1 between open and closed conformations shown by superposition of cryo-EM maps (open, containing caffeine, ATP, Ca^2+^: EMD-8376; closed, EGTA-only: EMD-8391). *Arrows* indicate the relative movements of the cytosolic shell, which includes a tilting motion, with parts closer to the symmetry axis moving “upward” and parts closer to the corners moving “downward.” Similar movements are observed for RyR1 structures that are in an activated state but with the pore region still closed. If similar changes are induced by conformational changes in Ca_V_1.1 (directly or indirectly), *e.g.*, by pushing downward on the corners of RyR1 or by pulling upward on RyR1 regions closer to the 4-fold symmetry axis, these would be predicted to activate RyR1. RyR1, ryanodine receptor 1; SR, sarcoplasmic reticulum.

RyRs are related to voltage-gated channels, with a transmembrane region containing at least six (or possibly eight) transmembrane helices (30, 31, 32). Four of these constitute a pseudo-VSD, and two contribute to the pore. A remarkable feature is a large cytosolic cap, which contains >80% of the entire protein. This cap is readily visible in thin sections of muscle fibers as electron-dense protrusions that span almost the entire gap between the T-tubular and SR membrane (33). Figure 3 outlines the overall structure of the RyR1, as obtained *via* cryo-EM studies (30, 32, 34). The bulk of the cytosolic cap is built up by α-solenoid regions and a series of globular domains. The size of the protein enables binding of a wide range of auxiliary proteins and small molecules (35). The various domains exposed on the cytosolic surface are attractive candidates for possible interactions with other components that allow the mechanical coupling between Ca_V_1.1 and RyR1 in skeletal muscle. The binding site for Ca^2+^ that is able to activate the channel is resolved (34) and is located next to a C-terminal Zn^2+^-finger domain immediately downstream of the last transmembrane segment.

RyRs are large allosteric proteins, such that conformational changes in regions far away from the pore can affect its opening. Cryo-EM studies have shown large movements of the cytosolic cap upon binding of activating ligands, such as Ca^2+^, ATP, and caffeine, and also by disease-associated mutations (34, 36, 37). A simplified description involves a “tilting” of the different subunits: the corners of the cytosolic cap move downward, whereas the center of the cytosolic surface moves upward and outward (Fig. 3). The implication for EC coupling is that, if conformational changes in Ca_V_1.1 can stimulate a similar tilting of the cytosolic cap, it would indirectly facilitate or trigger opening of the RyR1 pore.

The search for the mechanism that enables the coupling has been ongoing for several decades and included a dissection of various regions in Ca_V_1.1 and RyR1 that could form interactions with one another. One proposal included a direct interaction between the Ca_V_1.1 II-III loop and a domain known as SPRY2 in RyR1 (38), but another study failed to confirm this contact (39). Direct contacts have also been suggested from freeze-fracture studies, which showed a tetrad of Ca_V_1.1 channels juxtaposed to a single RyR1 (40). However, this does not preclude the presence of other small proteins that may link both channels. Although the field stagnated for a while, the appearance of STAC3 has spurred a new wave of investigations.

## STAC3 in excitation–contraction coupling

In 1996, Watanabe and coworkers cloned cDNA encoding a small 403-amino-acid protein from mouse brain (41). Sequence analysis revealed the presence of an SH3 domain and cysteine-rich region, and hence the protein was called “STAC” (Src homology three and cysteine rich domains). Its function remained unknown for many years, but in 2013 two independent groups discovered that STAC3, a different isoform, is a critical component in skeletal muscle EC coupling. Using a forward genetic screen in zebrafish, Horstick *et al.* (42) isolated an autosomal recessive mutant with severe motor behavior defects. These zebrafish die as larvae and defective motor behaviors manifest early in development. After ruling out malfunction in the nervous system, contractile machinery, triad anatomy, and calcium stores the authors noted reduced calcium transients in the muscles, indicative of a defect in EC coupling. Mapping the mutation to the gene encoding STAC3, they determined that their isolated mutant resulted in a functionally null protein that, owing to a premature stop codon, was not synthesized. Mutant rescue experiments successfully restored calcium transients with wildtype STAC3. Pull-down assays showed that STAC3 immunoprecipitates with both Ca_v_1.1 and RyR1, suggesting possible direct interactions. Similarly, Nelson *et al.* (43) found, through a knockout (KO) mouse model and primary myoblast culture, that STAC3 is required for EC coupling and localizes to the triad. STAC3-null mice are completely paralyzed and die at birth owing to asphyxia. They also found defects in muscle development, mass, and morphology. These findings led to renewed enthusiasm in the search for the elusive link between Ca_v_1.1 and RyR1.

In humans, STAC3 is encoded by a gene in chromosome 12q13-14; it is a 364-residue (41 kDa) soluble protein expressed predominantly in skeletal muscle (43, 44). Protein and mRNA profiles confirm there is no STAC3 expression in cardiac or smooth muscle, and since these do not display the mechanical coupling, this would confirm a role for STAC3 specifically in skeletal muscle EC coupling. A more detailed sequence analysis shows that it consists of an N-terminal poly-Glu region and a C1 domain linked *via* intrinsically disordered regions to tandem SH3 domains (41). Two other isoforms, STAC1 and STAC2, also exist and share high sequence identity with STAC3; they are expressed in a variety of tissues including the brain, reproductive system, and endocrine system (45).

The complexity of muscle tissue makes it hard to decipher which components are essential for EC coupling and which are only modulators. Recently, EC coupling was recapitulated in nonmuscle cells and required the heterologous expression of only five components: Ca_v_1.1, RyR1, Ca_V_β_1a_, STAC3, and a protein known as junctophilin (Fig. 1) (46). The latter is thought to organize the local membrane arrangement, by virtue of a C-terminal transmembrane helix, spanning the SR membrane, and an N-terminal MORN repeat domain that binds phospholipids in the T-tubular membrane (47). The Junctophilin 2 isoform was used in the study. This assembly of proteins is functional in tsA201 cells, with a depolarization of the plasma membrane resulting in Ca^2+^ release. Freeze-fracture electron microscopy confirms that a physical link is formed between the two calcium channels under these conditions wherein tetrads (corresponding to Ca_v_1.1) are aligned with the RyR homotetramers. As each of the five components was essential for recapitulating EC coupling, this confirms STAC3 as a fundamental component. However, it cannot be excluded that other proteins, natively expressed in tsA201 cells, are also involved in the process.

The link between Ca_V_1.1 and RyR1 is not unidirectional. The activation of RyR1, after depolarization of the plasma membrane, is also known as an “orthograde” signal. However, the presence of RyR1 also affects the properties of Ca_V_1.1: RyR1 quickens the activation kinetics and increases the amplitude of current through Ca_v_1.1, a phenomenon also known as “retrograde” coupling (5). Of interest, when all auxiliary subunits (β, α_2_δ, and STAC3) were coexpressed with Ca_V_1.1 in tsA201 cells, the currents were remarkably similar to those in myotubes, despite the absence of RyR1 (48). Myotubes in which STAC3 is knocked out display conductive properties nearly identical to dyspedic myotubes (lacking RyR1), implying that STAC3 is required also for retrograde coupling (49).

Having presented an overview of the EC coupling machinery in skeletal muscle, we will now discuss what is known to date regarding the structure, function, and interactions of the STAC domains. Table 1 summarizes the various proposed interactions of Ca_V_ channels with domains in STAC.

**Table 1:** Proposed interactions between STAC and Ca_v_ channels

Protein	Domain	Residue range	Binding partner	Functional significance	Expression system	Reference
STAC	C1 domain	93–150	Ca_v_1.2	Stable interaction, CDI	Dysgenic myotubes, tsA201 cells	(69)
	U-motif	174–191	Ca_v_1.2, Ca_v_1.3	CDI	HEK293 cells	(77)
	Linker region	171–289	Ca_v_1.2	CDI	tsA201 cells	(76)
	Tandem SH3	245–364	Ca_v_1.1 II-III loop	ECC	Purified protein	(56)
Ca_v_1.1	II-III loop	747–760 (minimal peptide)	STAC^a^ SH3 domains	ECC	Purified protein	(56)
	II-III loop	745–765	STAC^a^ SH3 domains	ECC	STAC3-null myotubes, tsA201 cells	(59)
Ca_v_1.2	Proximal CTD (IQ)	1689–1716	STAC^a^	CDI	Dysgenic myotubes	(78)
	Proximal CTD	1526–1858	STAC^a^	CDI	tsA201 cells	(76)
Ca_v_1.3	Proximal CTD (EF + pre-IQ)	1463–1593	STAC^a^	CDI	HEK293 cells	(77)
Ca_v_3.2	Distal NTD	51–100	STAC1	Trafficking	tsA201 cells	(67)

Abbreviations: CDI, calcium-dependent inactivation; ECC, excitation–contraction coupling.

All residues numbering is for the human isoform and the STAC numbering corresponds to STAC3.

a The interaction is shown for all isoforms.

## Domain architecture

**Figure 4:**
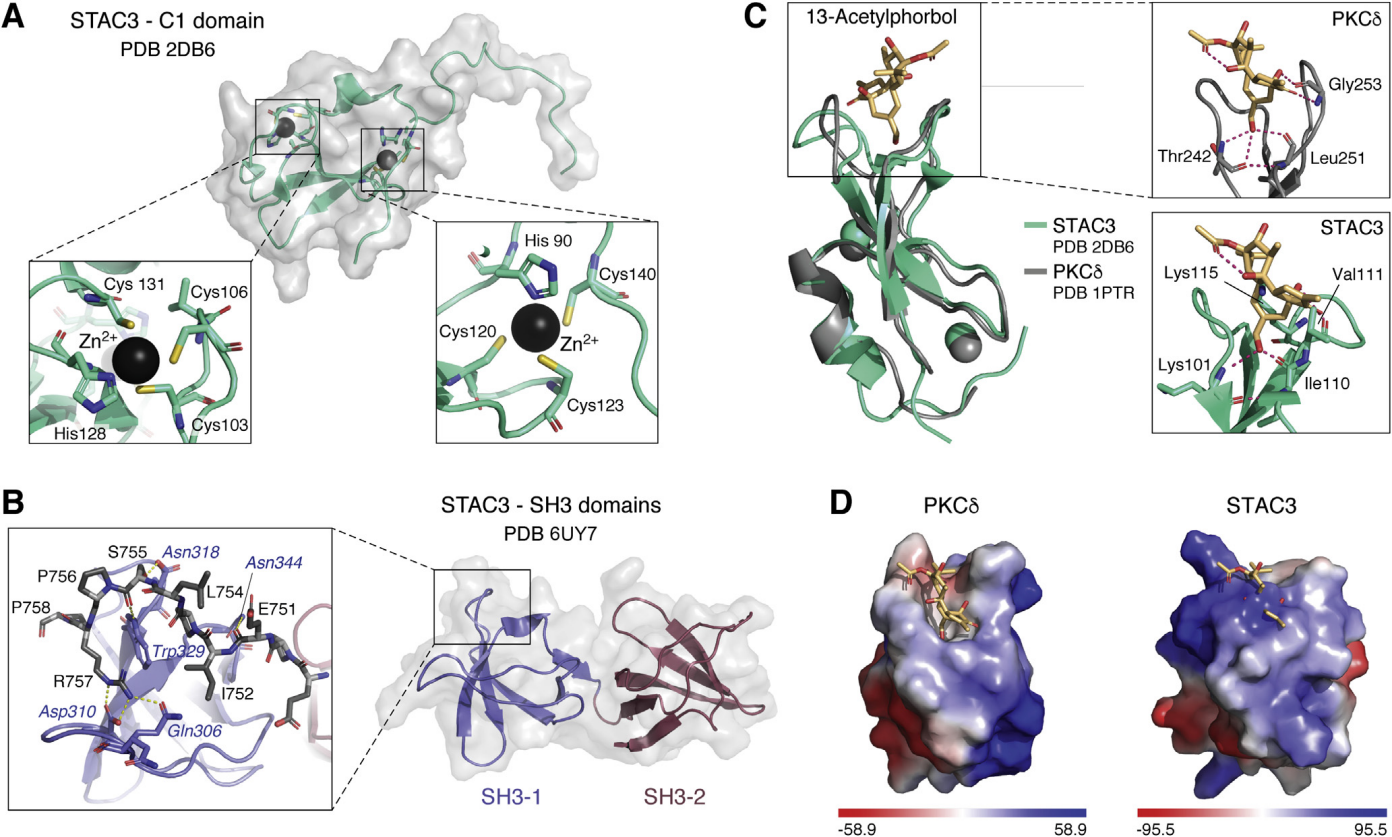
**Structures and lipid-binding properties of STAC domains.***A*, representative structure of the NMR ensemble for the STAC3 C1 domain (PDB: 2DB6). Two Zn^2+^ are each coordinated by three cysteines and one histidine, as indicated in the *insets*. *B*, crystal structure of the tandem SH3 domain of STAC3 (PDB: 6UY7). Each domain is composed of a five-stranded antiparallel beta sheet. The domains form a rigid interface by virtue of hydrophobic interactions. *Inset* shows the interactions of STAC2 with the Ca_v_1.1 II-III loop (PDB: 6B27). *C*, overlay of C1 domains of STAC3 (PDB ID: 2DB6) and PKCδ (PDB ID: 1PTR). For STAC3, the phorbol ester is shown aligned from the PKCδ structure. PKCδ is a “typical” C1 domain that binds to 13-acetylphorbol. The lipid binds within an activator-binding groove and forms hydrogen bonds with the backbone of T242, G253, and L152. In STAC3, this groove is occupied with the side chains of V111 and K115, which would clash with the lipid. Accordingly, lipid binding in STAC3 is unlikely without significant conformational changes. *D*, the electrostatic surface potential profiles of the C1 domains. The range for coloring is indicated below in k_b_Te_c_^−1^, where k_b_ is the Boltzmann constant, T is the absolute temperature in Kelvin, and e_c_ is the absolute value of the charge of a proton. In PKCδ part of the binding groove is formed by hydrophobic residues, whereas this region is largely positively charged in STAC3. However, in PKCδ positively charged residues do interact with negatively charged phospholipids at the membrane. PDB, Protein Data Bank.

STACs are composed of an N-terminal C1 domain linked *via* an intrinsically disordered region to two SH3 domains (41). C1 domains are typically lipid-binding modules originally described in protein kinase C (50). However, they also frequently act as protein–protein interaction motifs (51, 52, 53). An NMR structure is available for the STAC3 C1 domain (PBD ID 2DB6), although a description of this structure has not been published. The structure shows a typical C1 domain fold, with two Zn^2+^ ions bound. In each site, the Zn^2+^ is coordinated by three cysteines and one histidine (Fig. 4) (54). The region adjacent to the C1 domain, part of the NMR ensemble, is disordered. Secondary structure elements are limited to two antiparallel beta strands and a short alpha helix. In other proteins, C1 domains have been shown to be involved in lipid binding, namely, diacylglycerol or phorbol esters. Even among C1 domains with high sequence similarity, some are sensitive to lipids, whereas others (termed atypical) are not (50). Figure 4 shows the NMR structure of the PKCδC1B domain in complex with 13-acetylphorbol. Ligand binding does not induce significant conformational change in the protein (55). The lipid occupies a binding groove and hydrogens bonds the backbone of several residues. In STAC3, this groove is occupied by the sidechains of L115 and V111, which would clash with the ligand. Therefore, binding of phorbol esters to the STAC C1 domain would require substantial conformational changes. In addition, the electrostatic potential profiles of the proteins differ, with STAC3 having many positively charged residues surrounding the proposed binding pocket (Fig. 4). Although this makes the STAC3 C1 domain less likely to bind lipids, this remains to be verified experimentally.

SH3 domains are common protein-interaction modules that bind proline-rich segments containing a PxxP motif. We have recently solved high-resolution crystal structures of the tandem SH3 domains of all STAC isoforms (Fig. 4). Each domain is composed of a five-stranded anti-parallel beta sheet and short 3_10_ helix (56, 57). The two domains are connected by an unusually short linker of only five residues, and their interface is extensive with many hydrophobic interactions. As a result, the two SH3 domains form a rigid unit, rather than forming two beads on a flexible string. They can thus form one continuous interface with another protein. The fold of all three isoforms is very similar.

There is no structural information available for either the N terminus or linker region of STAC3, although they are predicted to be disordered based on sequence. Thus, the C1 and tandem SH3 regions are likely very mobile relative to one another.

## A critical interaction for EC coupling

**Figure 5:**
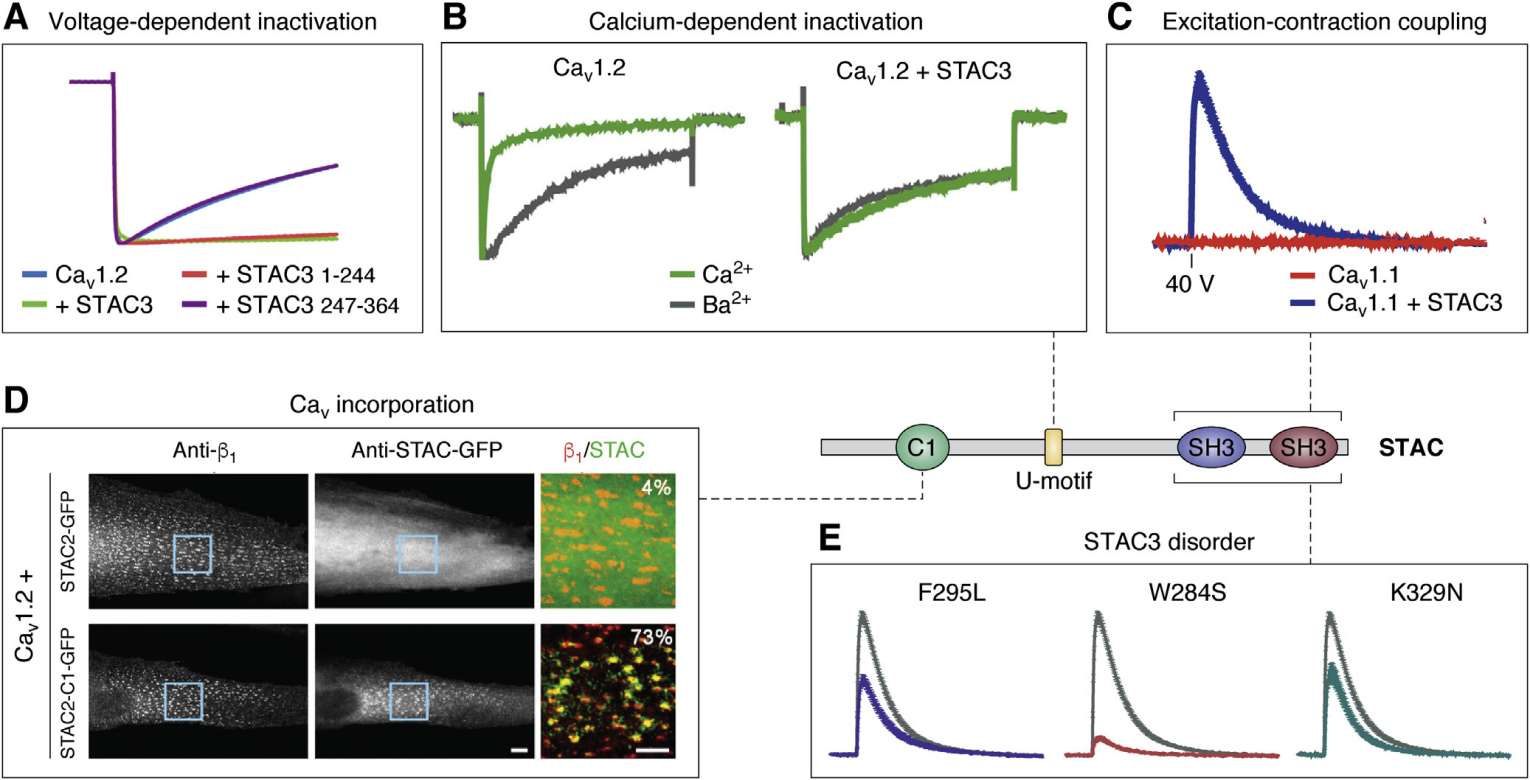
**The functional effects of STAC domains on voltage-gated calcium channels.***A*, STACs inhibit voltage-dependent inactivation in Ca_v_1.2. Normalized Ba^2+^ currents of oocytes expressing Ca_V_1.2 α1c in the presence and absence of the indicated STAC3 constructs. It is unknown which region is responsible for this effect, but the SH3 domains are not required (56). *B*, STACs inhibit calcium-dependent inactivation of L-type calcium channels. Normalized Ca^2+^ and Ba^2+^ currents at V_max_ of tsA201 cells expressing Ca_V_1.2. Difference in calcium-dependent inactivation *versus* voltage-dependent inactivation is quantified by using Ca^2+^ or Ba^2+^ as a charge carrier (78). A 22-amino-acid sequence termed the U-motif is sufficient to abolish calcium-dependent inactivation. *C*, STAC3 is a critical component of skeletal muscle excitation–contraction coupling. Double-KO (Ca_v_1.1/STAC3) myotubes loaded with Fluo-4 were reconstituted with GFP-Ca_v_1.1 and STAC3. Calcium transients were invoked *via* field stimulation with a 2-ms 40-V pulse. Cells lacking STAC3 are unable to produce Ca^2+^ transients (57). *D*, the C1 domain is responsible for stable incorporation of STAC into the Ca_v_1.1 complex. Ca_v_1.2 does not colocalize with STAC2 in dysgenic myotubes unless its C1 domain is replaced by that of STAC3 (69). *E*, mutations in STAC3 cause a congenital myopathy. Double-KO (Ca_v_1.1/STAC3) myotubes loaded with Fluo-4 were reconstituted with GFP-Ca_v_1.1 and STAC3. Calcium transients were invoked with a 2-ms 40-V pulse. The three variants shown are all in the SH3 domains and weaken binding to the Ca_v_1.1 II-III loop, impairing EC coupling calcium transients (57).

The cytoplasmic linker between repeats II and III of Ca_v_1.1 is a region long known to be critical for EC coupling (22, 23, 58). This linker region is sufficient to endow the cardiac isoform (Ca_v_1.2) with the ability to support mechanical coupling in dysgenic (Ca_v_1.1^−/−^) myotubes (22, 58). Through isothermal titration calorimetry we have shown that the proline-rich II-III loop binds to STAC proteins with low micromolar affinity (56). The “minimal motif” of Ca_v_1.1 for this interaction comprises residues 747 to 760, containing three key proline residues, although a longer region encompassing residues 728 to 778 has higher affinity, suggesting there are additional binding determinants outside the minimal motif. Using colocalization as an indication of interaction, all STAC isoforms bind the II-III loop of Ca_v_1.1 in tsA201 cells (59). Disrupting this interaction interferes with EC coupling and causes congenital myopathy (Fig. 5*E*) (56, 60).

We obtained a structure of the STAC2 SH3 domains in complex with a minimal peptide of the Ca_v_1.1 II-III loop (Fig. 4). Central to this interaction on the side of STAC2 is W329 (equivalent to W284 in STAC3); when substituted to a serine, the binding to Ca_v_1.1 is no longer detectable *via* isothermal titration calorimetry (56, 57). The first SH3 domain of STAC is the main contributor to binding; however, it alone has ~40-fold weaker binding compared with the tandem domains (56). The second SH3 domain contributes to binding through a water-mediated hydrogen bond, and likely also through general electrostatic interactions with negative charges on the II-III loop. However, this does not involve the area of this second SH3 domain that would form canonical interactions with proline-rich ligands. As this latter site remains unoccupied, it is thus free to potentially bind another protein. Molecular dynamics simulations predict that STAC3 binds the Ca_v_1.1 peptide in a nearly identical manner to STAC2, despite several differing residues at the interface for these isoforms (57).

Cryo-EM studies have revealed the structure of Ca_V_1.1 at greater than 3-Å resolution (61). However, owing to high intrinsic mobility, the cytosolic II-III loop and carboxy tail remain largely unresolved (Fig. 2). The next step would be a cryo-EM structure of STAC3 in complex with Ca_v_1.1, allowing insights into the allosteric regulation STAC3 may have on this calcium channel.

Having established STAC3 as an important auxiliary protein in the functional coupling between Ca_v_1.1 and RyR1 in skeletal muscle, we will delve into other diverse regulatory roles of STAC proteins discovered so far. To date, the roles have mostly focused on regulation of L-type Ca_V_ channels.

## Functional effects of STACs on Ca_v_ channels

### Channel trafficking

One unique property of Ca_v_1.1 among Ca_V_s is that it does not express well in nonmuscle cells (62, 63, 64). Although coexpression of Ca_v_1.1 with the β and α_2_δ subunits gives low baseline membrane expression, this complex is mostly retained in the endoplasmic reticulum of heterologous systems (48, 65), and this has hampered systematic functional investigation of Ca_V_1.1 *via* heterologous expression. However, when coexpressed also with STAC3, Ca_v_1.1 robustly traffics to the plasma membrane (60). This complex shows conductive properties similar to those in myotubes. Conversely, myotubes and myofibers that lack STAC3 show reduced expression of both Ca_V_1.1 and RyR1 (43, 49, 66).

Appreciable membrane expression of Ca_v_1.1 can also be achieved when expressed with the γ-subunit in addition to β and α_2_δ, although this complex lacks normal Ca^2+^ currents (48). Calmodulin (CaM), which interacts with the carboxy tail of Ca_v_s, can also rescue trafficking deficits for Ca_v_1.1 (65). Truncation or replacement of the C-terminal domain (CTD) of the channel also increases surface expression. As such, STAC3 and CaM may mask a retention signal in the CTD, albeit by different mechanisms. Overall, STAC3 facilitates, but is not required for, trafficking of Ca_v_1.1 to the plasma membrane. Analogously, STAC1 promotes surface expression of T-type Ca_v_3.2 channels (67). This regulation depends on STAC1 binding the distal N-terminal domain. As experiments with Ca_V_1.1 in heterologous expression systems were previously impractical, the ability of STAC to enhance expression now provides more opportunity for structure–function studies (46, 68).

Originally, experiments with fluorescently tagged STAC3 in dysgenic (Ca_v_1.1 ^−/−^) and STAC3-KO myotubes indicated that Ca_v_1.1 is required for triad targeting of STAC3 (48, 69). That is, in the absence of Ca_v_1.1, transfected STAC3 was diffusely spread through the cytoplasm. Conversely, a later report used an isoform-specific antibody to label endogenous STAC3 in myotubes and muscle fibers providing evidence to the contrary (70). Immunostaining suggests that STAC3 colocalizes with RyR1 in the absence of Ca_v_1.1. The authors propose a competition model, where association of endogenous STAC3 is sufficiently strong in the triad as to prevent replacement with tagged STAC3. Ca_v_1.1 presents additional binding sites, which can be occupied by exogenous protein. The recent generation of a double Ca_v_1.1-STAC3 KO myotube cell line may provide further insight into this tentative model (57).

Armed with an improved understanding of how STAC proteins help transport of Ca_v_s to the plasma membrane, we will describe their effect on the channels once they arrive at the plasma membrane, namely, the influence on calcium currents.

### Calcium-dependent inactivation

Precise control of the current through voltage-gated calcium channels is critical for maintaining the proper Ca^2+^ signals in excitable cells. As such, negative feedback mechanisms exist, including voltage- and calcium-dependent inactivation (VDI and CDI), which readily reduce the currents (13). The mechanisms of these processes are not fully understood, but the ubiquitous calcium-sensing protein CaM is a critical regulator of CDI (71, 72, 73). CaM interacts with the IQ domain in the carboxy terminus of Ca_v_s, as well as with an N-terminal region known as NSCaTE, present in some isoforms (74). In electrophysiology experiments, CDI is observed as an enhanced decay in currents when Ca^2+^ is used as a charge carrier *versus* Ba^2+^ (Fig. 5*B*). Several studies have demonstrated that STAC proteins inhibit both types of inactivation in L-type channels, although the effect on CDI has been investigated more thoroughly (Fig. 5, *A* and *B*) (56, 75). STACs do not appear to have any effect on calcium-dependent inactivation or calcium-dependent facilitation of non-L-type channels (Ca_v_2.1 or Ca_v_2.3) or Na_v_1.4 (76, 77).

Whole-cell calcium currents in tsA201 cells reveal that coexpression with STAC3 substantially reduces CDI of Ca_v_1.2 (Fig. 5*B*) (48). This effect of STAC proteins in general is confirmed in *Xenopus oocytes*, rat hippocampal neurons, and HEK293 cells; it holds true also for Ca_v_1.3 and Ca_v_1.4 (56, 76, 77). However, inhibition by STAC1 and STAC2 in tsA201 cells depends on the calcium buffering conditions: reduction in CDI is considerably weaker under 0.5 mM EGTA than under 10 mM EGTA (78). Mechanistically, single-channel recordings show that STAC-bound channels preferentially adopt a high open probability mode (77).

Colocalization and electrophysiology experiments with Ca_v_1.2/Ca_v_2.1 chimeras in dysgenic cells suggest the proximal CTD is necessary for regulation by STACs (78). This region contains the IQ motif, known to bind CaM (71, 73). STAC3 fails to abolish CDI when Ca_v_1.2 harbors the IQ domain of Ca_v_2.1. Indeed, the key residues for STAC association overlap with those for CaM binding, suggesting that STAC may block CaM regulation. In contrast, using FRET 2-hybrid assays, Niu *et al.* (77) found that STACs have a 10-fold higher affinity for the EF-hand and pre-IQ motif than the IQ domain. A fusion channel with CaM tethered to the C terminus retains STAC inhibition, implying STACs are not competitive inhibitors of CaM. Finally, using fragments of Ca_v_1.2 tagged with a surface-targeting peptide in tsA201 cells, Polster *et al.* (76) determined that multiple regions of the carboxy tail are important for STAC colocalization. However, the Pearson’s coefficient was similar with and without the IQ domain, arguing against its importance.

**Figure 6:**
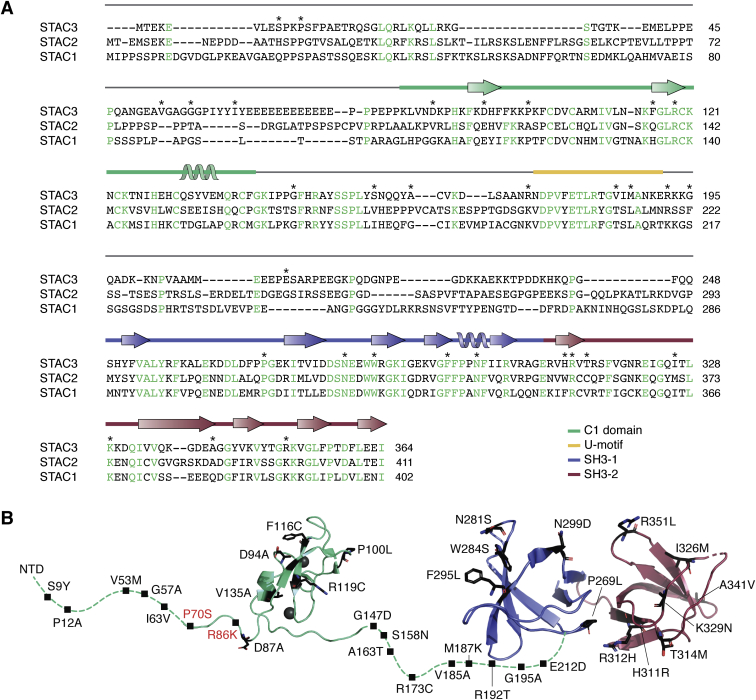
**Sequence and variants of STAC proteins.***A*, sequence alignment of STAC isoforms. Conserved residues are indicated in *green*. The secondary structure (corresponding to STAC3) is shown above the sequence. The locations for reported sequence variants in STAC3, possibly linked to disease, are indicated with ∗. *B*, the 3D architecture of STAC3, with disease-associated variants. Residues with variants reported in the ClinVar database are indicated—*black* represents STAC3 variants and *red* represents STAC2 variants.

Also controversial are the corresponding regions of STAC important for CDI regulation. One consistent consensus is that the SH3 domains are dispensable for this function. Two independent studies agree that the disordered linker region between the C1 and SH3 domains is sufficient to abolish CDI, whereas the other isolated domains cannot (76, 77). One study specifically identified a 22-residue “U-motif” responsible for CDI suppression (77). This peptide was able to disrupt CDI when applied to ventricular myocytes. The linker region is poorly conserved between STAC isoforms (Fig. 6*A*), despite the ability of all to regulate CDI (albeit to different degrees). However, several residues within the U-motif appear conserved (77). Alternatively, another study has implied the importance of the C1 domain instead (78). One possibility is that the U-motif is the critical element that mediates the suppression, whereas the C1 domain can further stabilize the complex (Fig. 5*D*).

Using fluorescence recovery after photobleaching and chimeric experiments, Campiglio *et al.* (69) showed that STAC3 is stably incorporated into the Ca_v_1.1 complex in dysgenic myotubes. Its turnover rate is comparable with that of Ca_v_β. In this system, the interaction is isoform specific on the side of STAC: STAC3 colocalizes with both Ca_v_1.1 and Ca_v_1.2, whereas STAC1 colocalizes only with Ca_v_1.2 and STAC2 only poorly with Ca_V_1.2. Absence of STAC2 binding allowed the authors to home in on the responsible domain by systematically replacing domains of STAC2 with those of STAC3. They deduced that the C1 domain is required for the stable interaction (Fig. 5*C*) and identified two critical residues (V104 and Y133) that likely form a protein-binding pocket. However, the isolated C1 domain cannot colocalize with Ca_v_1.2 suggesting that other regions of the protein also facilitate this interaction. On the contrary, in STAC3-null myotubes and tsA201 cells, STAC1 and STAC2 do colocalize with Ca_v_1.1. Dysgenic myotubes contain endogenous STAC3, which may compete with the other STAC isoforms. In STAC3-absent systems the C1 domain appears expendable for colocalization. Accordingly, multiple regions are likely responsible for STAC association with Ca_v_s; these may diverge between the Ca_v_ isoforms.

Of interest, the ability of STAC3 to suppress CDI through interactions with the EF-hand motif is reminiscent of fibroblast growth factor homologous factor proteins, which can abolish CDI of Na_V_1.4 (77). These fibroblast growth factor homologous factor proteins reorient the EF-hand domain relative to the IQ domain (79, 80, 81, 82, 83), possibly interfering with the ability of Ca^2+^/CaM to bind to the IQ domain (81). In a clever experiment, an artificial Ca_V_1.3 channel was engineered, containing a binding site for an SH3 domain from the adaptor protein Mona, just upstream of the IQ domain. Addition of this SH3 domain abolished CDI, showing that binding of proteins to the EF-hand domain and surrounding regions is a general mechanism through which CDI can be modulated (77). These parallel findings further stress that interactions between STAC3 and the CTD of L-type Ca_V_ channels have the ability to eliminate CDI.

STAC proteins thus function as potent inhibitors of the negative feedback mechanisms of Ca_v_s. As the mechanism of CDI and VDI in voltage-gated calcium channels is incompletely understood from a structural viewpoint, further research is needed to fully understand the mechanism through which STAC interferes with it. Based on the cryo-EM structure of Ca_v_1.1, the II-III loop would be adjacent to the C-terminal region in three-dimensional space, implying steric compatibility for interactions of the SH3 domains, C1 domain, and the U-motif with their proposed partners. In adult skeletal muscle, current activation through Ca_v_1.1 requires 30 mV stronger depolarization than for EC coupling. Therefore, its primary function is not as a calcium channel, but rather as a voltage sensor. However, calcium currents may play a role in muscle differentiation and prolonged activity (84). In addition, it remains controversial whether Ca_v_1.1 experiences CDI in a native physiological setting. Accordingly, it is difficult to speculate about the effect that inhibition of CDI by STAC3 may have in skeletal muscle. However, L-type calcium currents in a variety of neuronal cell types are prolonged and slowly inactivating (85, 86, 87). Diverse Ca^2+^-dependent processes like gene regulation and synaptic transmission may be affected by such Ca_v_ modulation.

### Muscle differentiation

STAC3 is implicated in several studies as a regulator of skeletal muscle differentiation. Deletion of STAC3 from postnatal mice suggest its importance for skeletal muscle growth and fiber-type composition (88, 89). STAC3 deletion inhibited the transition from type I to type II fibers in fast twitch muscle. Depleted myofibers had greater SR-calcium release than controls. However, some of these results may be explained by lack of muscle activity affecting muscle size and fiber type (90). Separate studies have looked at the effect of STAC3 in differentiating C2C12 myoblasts, with differing conclusions. Ge *et al.* (91) found that the presence of STAC3 decreases the number of nuclei per myotube and reduces expression of myogenic markers, suggesting that STAC3 inhibits muscle differentiation. Similar results were found in bovine satellite cells (92). Conversely, Bower *et al.* (93) suggest that STAC3 is necessary for muscle differentiation, finding that knockdown *via* RNA interference inhibits myotube formation. Additional research is needed to reconcile these inconsistencies.

## Does STAC3 interact with RyR1?

When STAC3 emerged as an essential component in EC coupling, the possibility arose that it could physically link Ca_V_1.1 and RyR1 in skeletal muscle, by interacting with both channels simultaneously. Although the interactions between STAC3 and Ca_V_1.1 are now well established and functionally verified, there is currently no unambiguous evidence for an interaction between STAC3 and RyR1. Although STAC3 is critical for the coupling, one possibility is that STAC3 alters the conformation of Ca_V_1.1, including the II-III loop, thus stimulating direct interactions between Ca_V_1.1 and RyR1. Another alternative is that STAC3 may recruit another protein, which then in turn interacts with RyR1.

There is indirect evidence that STAC3 may interact with RyR1, although this is inconclusive. For example, coimmunoprecipitation studies showed that RyR1 can pull down STAC3 and vice versa in zebrafish muscle (42). However, this is still compatible with an indirect interaction. A study with dysgenic myotubes, which lack Ca_V_1.1, showed that heterologously expressed STAC3 was no longer incorporating into triads, in contrast with wildtype or dyspedic myotubes (the latter lacking RyR1) (48, 69). Although this solidifies Ca_v_1.1 as a main binding partner for STAC3, it does not exclude the possibility that STAC3 can still bind RyR1, albeit weakly. Another study with an antibody against endogenous STAC3 did show STAC3 clustering in the triads of dysgenic myotubes (70). One possibility is that this represents an additional STAC3-binding site in triads, which is not displaced with heterologously expressed STAC3.

Regardless of whether STAC3 forms interactions with RyR1, it is clear that any such interaction would be very weak, similar to the entire EC-coupling complex. It is conceivable that multiple weak interaction points exist, for example, between RyR1 and Ca_V_β1, RyR1 and STAC3, or RyR1 and the Ca_V_1.1 II-III loop, but none of them is strong enough to be easily detected. Indeed, both RyR1 and Ca_V_1.1 can be routinely purified from skeletal muscle for cryogenic-electron microscopy studies, but neither seems to copurify with significant amounts of the other channel (15, 30, 32). This also underscores the inherent limitation with traditional biochemical preparations: as one of the first steps to purify membrane proteins involves detergent solubilization, the intricate triad membrane system is immediately disrupted, thus eliminating the high effective concentration of these proteins *in situ*, with the interactions now too weak to hold the complex together. Subtomogram averaging of EC coupling complexes *in situ* provide the most likely avenue to solve the entire EC coupling puzzle.

## Disease

STAC3 is the target for mutations causing the rare neuromuscular disease Native American myopathy (NAM) (42, 60). NAM is an autosomal recessive disease (94, 95, 96, 97). Cases were first identified in members of the Lumbee Tribe of North Carolina; the prevalence within this community is difficult to estimate because of their cultural isolation (98). Since then, multiple STAC3 mutations, including the founder W284S mutation, have been identified in patients worldwide, so the name NAM is now a misnomer. We propose to use the term “STAC3 disorder” instead, which we will use from here on. Symptoms include short stature, muscle weakness, delayed motor development, cleft palate, scoliosis, and susceptibility to malignant hyperthermia (MH) (Table 2) (99). The last one is a disorder characterized by a hypermetabolic response upon administration of volatile anesthetics during surgery or muscle relaxants like succinylcholine (100). If untreated with dantrolene, MH can be fatal. It typically associates with mutations in RyR1, so its association with STAC3 further highlights the fact that these proteins are part of a larger complex. Many of the STAC3 disorder symptoms necessitate corrective surgery, which can then trigger MH responses, leading to several instances of severe MH-induced disability in patients. In fact, the first case of STAC3 disorder was reported based on an MH episode in a 3-month-old infant undergoing feeding gastronomy surgery owing to her difficulty swallowing (101). There have been several cases where STAC3 disorder is misdiagnosed owing to lack of formal diagnostic criteria, leading to unsuspected adverse reactions under general anesthesia (94, 99, 102). It is estimated that approximately one-third of patients die before the age of 18 years, prevalently from MH episodes or respiratory failure (98). There is currently no cure or treatment for this severely debilitating disease. Prevention of fatal episodes may come with genetic testing for accurate diagnosis and improved understanding of the molecular mechanisms of disease.

**Table 3:** Interaction partners of STAC proteins identified by at least two independent proteomics screens or two experimental methods

Isoform	Binding partner	Functional role	Assay	Reference
STAC1	YWHAE	14-3-3 protein	AC	(107, 116)
	ANKLE2	Mitotic nuclear envelope reassembly, brain development	AC	(116, 117)
	KANK2	Transcriptional regulation	2H	(118, 119)
	NCKIPSD	Stress fiber formation, angiogenesis	2H	(119, 120)
	YWHAZ	14-3-3 protein	AC	(116, 117)
	KIAA1958	Uncharacterized	2H	(118, 119)
	LZTS2	Microtubule regulator; Wnt signaling	2H	(118, 119)
	PARP2	DNA repair	PA, ES	(121)
STAC3	ZCCHC10	Uncharacterized	2H	(118, 119, 122)
	CSNK2A1	Serine/threonine-protein kinase complex subunit	2H	(118, 119, 123)
	PPARA	Transcription factor	2H	(119, 122)
	FAM133A	Uncharacterized	2H	(118, 119)
	EAF1	Transcriptional transactivator	2H	(119, 124)
	ENKD1	Uncharacterized	2H	(119, 124)
	C1ORF35	Uncharacterized	2H	(119, 124)
	WDR54	ERK signaling pathway	AC	(116, 117)
	GTF2F1	Transcription initiation factor	AC	(116, 117)
	SREK1IP1	Splicing regulator	2H	(118, 119)

Abbreviations: 2H, two hybrid assay; AC, affinity capture; ES, enzymatic study; PA, protein array; RS, reconstituted complex.

Data curated from the BioGrid, HuRT, IntAct, and UniProt databases.

The most commonly reported mutation is a tryptophan to serine substitution at residue 284 in the first SH3 domain of STAC3 (42). This residue is central to the interaction with the proline-rich II-III loop of Ca_v_1.1, forming a hydrogen bond and cation-pi interactions with the peptide (Fig. 4) (56). Several other variants are reported in clinical databases (Fig. 6), but the clinical significance and severity is unclear from the available data. We have shown that W284S, F295L, and K329N affect EC coupling by weakening the interaction with the II-III loop (Fig. 5*E*) (57). However, these variants did not have significant effects on the structure or stability of the SH3 domains. Of interest, H311R in the second SH3 domain does not reduce binding to the II-III loop, and it also affects EC coupling (57). This suggests that this region may interact with another triad protein, such as RyR1. Myofibers harboring the W284S mutation have reduced Ca_v_1.1 expression and rightshifted voltage-gated Ca^2+^ release and release significantly more Ca^2+^ in response to caffeine than wildtype fibers (60, 65). The latter is a result of increased luminal SR Ca^2+^.

A *de novo* mutation was reported in one gene copy of a Turkish patient that introduced a premature stop codon in the first SH3 domain (K288stop) (95). EC coupling is expected to be abolished with this mutant as it relies on the integrity of both SH3 domains. We also examined a variant in which four residues within a beta strand of the second SH3 domain are deleted by aberrant splicing (96). We found *via* size exclusion chromatography that this protein was aggregated, implying it is misfolded, likely leading to the disease phenotype (57).

Several variants are within or flanking the U-motif (R173C, V185A, M187K, R192T); these may potentially interfere with STAC’s regulation of CDI. Of the variants in the C1 domain, none involve Zn^2+^-coordinating residues. P100 is located within the typical lipid-binding region of C1 domains, but as described above it seems unlikely that STAC3 binds lipids, putting to question the effects this mutation would have.

As STAC3 interacts with Ca_V_1.1, one could expect corresponding mutations in Ca_v_1.1 that would give rise to a phenotype similar to STAC3 disorder. Although Ca_V_1.1 is also a known target for MH (103), so far, no mutations have been identified in the STAC3-binding site within the II-III loop. Such mutations have been found in Ca_V_1.2: several patients with type 8 long QT syndrome harbor mutations in a short segment of the Ca_v_1.2 II-III loop, which maps directly to the STAC-binding site (104). Unexpectedly, these patients experience arrhythmic symptoms and sudden death. Although these residues would affect STAC binding in neuronal Ca_V_1.2, STACs are reportedly absent from cardiac tissue, so the cardiac phenotype could not easily be explained from this observation. Possibly, another SH3-domain-containing protein may associate with this region in cardiac tissue, and the mutations affect this interaction.

Although most STAC protein variants map to STAC3, a *de novo* missense mutation in STAC2 is implicated in childhood-onset schizophrenia. Of interest, variants in RyR2 have also been associated with this disorder (105, 106). In addition, another mutation in STAC2 is associated with severe myopia. Both variants are located in the N-terminal region, between a poly-proline sequence and the C1 domain (Fig. 6). These variants highlight the role of STAC proteins in other tissues, but where they have been less studied.

Thus overall, STAC proteins have also emerged as targets for various disorders. Although a subset of the sequence variants in the STAC3 tandem SH3 domains can be readily understood in terms of affecting interactions with the Ca_V_1.1 II-III loop, a complete understanding of variants in other regions will only be possible with a more complete picture of the various additional protein–protein interactions in which STACs are involved. In the next section, we discuss the various additional putative binding partners and signaling pathways involving STAC proteins.

## STAC proteins in other signaling pathways

Although STAC proteins are now firmly established as modulators for L-type Ca_V_ channels and critical elements for muscle EC coupling, emerging evidence suggests that they play roles in other signaling pathways. Several proteomics screens have identified potential interacting partners of STAC proteins, as summarized in Table 3. Of note, STAC proteins have putative roles in several signaling pathways. STAC1 interacts with 14-3-3 proteins, a family expressed abundantly in the central nervous system and involved in cell proliferation and apoptosis (107). A novel splice variant of STAC1 is implicated in senescence in human mammary fibroblasts, which limits the proliferation potential of the cell (108). However, the potential mechanism of its involvement is unknown. STAC2 interferes with the RANK signaling complex formation to negatively regulate osteoclast formation (109). This isoform has also been identified as a differentially expressed gene in multiple studies of breast cancer, a tissue in which it is abundantly expressed (110, 111). Although STAC3 was originally believed to be expressed solely in skeletal muscle tissue, a recent study has found it is also expressed in Leydig cells of the male reproductive system (112). Here, STAC3 depletion appears to disrupt steroidogenesis by inducing mitochondrial dysfunction. Although many of these interactions remain to be validated through quantitative binding assays, the role of STACs as regulatory proteins may thus extend well beyond ion channels. A word of caution is needed, however, in regards to large screens that have aimed to identify the interactome of STAC proteins. As STAC proteins contain large stretches of positively charged residues, cell lysis can result in nonspecific binding to nucleic acid, which in turn can interact with a host of other proteins. These may lead to false-positive hits. Indeed, our own purification procedures of STAC proteins have shown that nucleic acid readily copurifies, and stringent washing procedures are required to remove these for any crystallographic or quantitative binding assay.

**Table 2:** Clinical data for STAC3 disorder cases found in the literature

Reference	Descent	Cases	Symptoms	MH episodes	STAC3 variant
(94)	Qatari	2	Hypotonia, myopathic facies, generalized weakness, ptosis, cleft palate, growth delay, kyphoscoliosis, asymmetric hip position, long face, swallowing difficulty	2/2	W284S
	Puerto Rican	2	Facial & generalized weakness, cleft palate, hypotonia, respiratory impairment, hearing loss	1/2	W284S, Leu255IlefsX58
(95)	Turkish	1	Congenital muscle weakness, scoliosis, respiratory impairment, ptosis, short stature, delayed motor development, oral hypotonia	No	K288∗, c.432+4A>T
(97)	N/A	1	Hypotonia, respiratory impairment, cleft palate, club feet, adducted thumbed, hip dysplasia, muscle weakness, scoliosis	Yes	W284S
(96)	African, Middle Eastern, Comorian, South American	17	(Variable) hypotonia, talipes, contractures, cleft palate, respiratory impairment, limb weakness, ptosis, facial weakness, muscle atrophy, scoliosis, spinal rigidity, hearing loss	9/17	W284S
	Afro-Caribbean	1	Multiple contractures, hypotonia, respiratory impairment, cleft palate, elevated hemi-diaphragm, facial weakness, scoliosis, ptosis, myopathic face	Yes	W284S, Δ333IVVQ336
(98)	Lumbee	14	(Variable) delayed motor development, myopathic face, ptosis, downturned mouth, cleft palate, micrognathia, congenital joint contractures, scoliosis 36% mortality rate	4/14	N/A
(42)	Lumbee	5	Not indicated in publication	N/A	W284S

Abbreviation: MH, malignant hyperthermia.

If only one allele is listed, the variant is homozygous. If two are shown, each corresponds to one gene copy.

mRNA expression profiles indicate that STAC1 and STAC2 are also highly expressed in some nonexcitable cells. Knockout mice of STAC1 and STAC2 are available and in both cases are homozygous viable. The International Mouse Phenotyping Consortium reports no significant phenotypes for either model, although the data are not complete (113). Owing to overlap of expression profiles and high sequence similarity, it is also possible that one STAC isoform could compensate for loss of the other in tissues in which they are both expressed. Hence, a double knockout of both STAC1 and STAC2 may be required to elucidate their physiological roles.

STAC proteins are an emergent class of ion channel regulators. Although they were originally discovered in neurons, their function in muscle has been the focus of most literature to date, leaving the functional consequences of neuronal STACs largely unexplored. STAC1 and STAC2 are abundantly expressed in the central nervous system; they affect VDI and CDI of L-type voltage-gated calcium channels expressed in the same regions. Inhibition of channel inactivation leads to prolonged calcium currents and may affect diverse downstream calcium signaling processes, such as excitation–secretion and excitation-transcription coupling. Thus, STACs may affect these processes, although this remains to be shown. DStac, homologous to mammalian STAC genes, is expressed in muscles and a subset of neurons in *Drosophila melanogaster*. As in mammals and zebrafish, it is required for locomotion. In addition, it was shown to regulate the release of the exogenously expressed neuropeptide Dilp-2 (insulin-like peptide 2) from motor neuron boutons (114). Specifically, release of Dilp2-GFP was decreased in boutons with DStac knockdown, or where the SH3 domain had been deleted *via* CRISPR-Cas9. This regulation likely arises through an interaction with the major L-type Ca_v_ in these neurons (Dmca1), whereby DStac affects the calcium current that triggers neuropeptide release. Proctolin is the neuropeptide endogenously expressed in these boutons, and its release increases muscle contractions. Another neuropeptide, pigment-dispersing factor, regulates circadian rhythm in *Drosophila*, suggesting that neuropeptide release could link previous findings that DStac is required for normal circadian rhythm (115). These findings provide the first known function for STAC proteins in neurons.

## Conclusion

STAC3 is a critical component of skeletal muscle EC coupling and holds potential as the auxiliary protein linking Ca_v_1.1 and RyR1. STAC proteins have a myriad of regulatory roles for voltage-gated calcium channels in which they mediate functional interactions, trafficking, and channel inactivation. Many details of STAC function remain contentious: still entangled are the roles of the various domains and yet undiscovered are additional interacting partners.

From a structural biology viewpoint, obvious directions include cryo-EM structures of complexes of Ca_V_1.1 + STAC3, or between RyR1 + STAC3. As either membrane protein can be readily purified from muscle tissue, and since resolutions near 3 Å can be obtained, various details of STAC3 binding could be revealed. However, the possibility exists, especially for Ca_V_1.1, that the binding only involves very flexible regions. Indeed, the II-III loop is invisible in cryo-EM reconstructions of Ca_V_1.1 to date, so this would only be successful if STAC3 binding immobilizes this element by virtue of additional contact points. In the case of RyR1, an intrinsic problem may be in the low affinity, and cross-linking may therefore need to be considered. In the ideal scenario, the entire EC coupling complex would be captured, but this is unlikely to happen using standard cryo-EM methods, as the membrane system of the triad will need to remain intact. As such, cryo-electron tomography, along with subtomogram averaging will be a worthwhile endeavor.

Given the ability of STAC1 and STAC2 to regulate CDI of L-type Ca_V_s in the central nervous system, it will be of particular interest to see whether this affects various downstream Ca^2+^-dependent processes. Finally, with the emerging list of STAC interactors, the door is open for quantitative and structural investigations, which will help validate the proposed binders. Although the roles of these eclectic adaptor proteins had long remained elusive, we are slowly beginning to unravel their biological importance.

## Conflicts of interest

The authors declare that they have no conflicts of interest with the contents of this article.

## References

[1] Endo M., Tanaka M., Ogawa Y. (1970). Calcium induced release of calcium from the sarcoplasmic reticulum of skinned skeletal muscle fibres. Nature.

[2] Armstrong C.M., Bezanilla F.M., Horowicz P. (1972). Twitches in the presence of ethylene glycol bis( -aminoethyl ether)-N,N’-tetracetic acid. Biochim. Biophys. Acta.

[3] Schredelseker J., Shrivastav M., Dayal A., Grabner M. (2010). Non-Ca2+-conducting Ca2+ channels in fish skeletal muscle excitation-contraction coupling. Proc. Natl. Acad. Sci. U. S. A..

[4] Dirksen R.T., Beam K.G. (1999). Role of calcium permeation in dihydropyridine receptor function insights into channel gating and excitation-contraction coupling. J. Gen. Physiol..

[5] Nakai J., Dirksen R.T., Nguyen H.T., Pessah I.N., Beam K.G., Allen P.D. (1996). Enhanced dihydropyridine receptor channel activity in the presence of ryanodine receptor. Nature.

[6] Avila G., Dirksen R.T. (2000). Functional impact of the ryanodine receptor on the skeletal muscle L-type Ca(2+) channel. J. Gen. Physiol..

[7] Chavis P., Fagni L., Lansman J.B., Bockaert J. (1996). Functional coupling between ryanodine receptors and L-type calcium channels in neurons. Nature.

[8] Usachev Y.M., Thayer S.A. (1997). All-or-none Ca2+ release from intracellular stores triggered by Ca2+ influx through voltage-gated Ca2+ channels in rat sensory neurons. J. Neurosci..

[9] Berrout J., Isokawa M. (2009). Homeostatic and stimulus-induced coupling of the L-type Ca2+ channel to the ryanodine receptor in the hippocampal neuron in slices. Cell Calcium.

[10] De Crescenzo V., Fogarty K.E., Zhuge R., Tuft R.A., Lifshitz L.M., Carmichael J., Bellvé K.D., Baker S.P., Zissimopoulos S., Lai F.A., Lemos J.R., Walsh J.V.J. (2006). Dihydropyridine receptors and type 1 ryanodine receptors constitute the molecular machinery for voltage-induced Ca2+ release in nerve terminals. J. Neurosci..

[11] Mouton J., Marty I., Villaz M., Feltz A., Maulet Y. (2001). Molecular interaction of dihydropyridine receptors with type-1 ryanodine receptors in rat brain. Biochem. J..

[12] Hopp S.C., D’Angelo H.M., Royer S.E., Kaercher R.M., Crockett A.M., Adzovic L., Wenk G.L. (2015). Calcium dysregulation via L-type voltage-dependent calcium channels and ryanodine receptors underlies memory deficits and synaptic dysfunction during chronic neuroinflammation. J. Neuroinflammation.

[13] Zamponi G.W., Striessnig J., Koschak A., Dolphin A.C. (2015). The physiology, pathology, and pharmacology of voltage-gated calcium channels and their future therapeutic potential. Pharmacol. Rev..

[14] Zühlke R.D., Pitt G.S., Tsien R.W., Reuter H. (2000). Ca2+-sensitive inactivation and facilitation of L-type Ca2+ channels both depend on specific amino acid residues in a consensus calmodulin-binding motif in the(alpha)1C subunit. J. Biol. Chem..

[15] Wu J., Yan Z., Li Z., Qian X., Lu S., Dong M., Zhou Q., Yan N. (2016). Structure of the voltage-gated calcium channel Ca v 1.1 at 3.6 Å resolution. Nature.

[16] Van Petegem F., Duderstadt K.E., Clark K.A., Wang M., Minor D.L.J. (2008). Alanine-scanning mutagenesis defines a conserved energetic hotspot in the CaValpha1 AID-CaVbeta interaction site that is critical for channel modulation. Structure.

[17] Van Petegem F., Clark K.A., Chatelain F.C., Minor D.L.J. (2004). Structure of a complex between a voltage-gated calcium channel beta-subunit and an alpha-subunit domain. Nature.

[18] Opatowsky Y., Chen C.-C., Campbell K.P., Hirsch J.A. (2004). Structural analysis of the voltage-dependent calcium channel beta subunit functional core and its complex with the alpha 1 interaction domain. Neuron.

[19] Chen Y.-H., Li M.-H., Zhang Y., He L.-L., Yamada Y., Fitzmaurice A., Shen Y., Zhang H., Tong L., Yang J. (2004). Structural basis of the alpha1-beta subunit interaction of voltage-gated Ca2+ channels. Nature.

[20] Norris N.C., Joseph S., Aditya S., Karunasekara Y., Board P.G., Dulhunty A.F., Oakley A.J., Casarotto M.G. (2017). Structural and biophysical analyses of the skeletal dihydropyridine receptor β subunit β_1a_ reveal critical roles of domain interactions for stability. J. Biol. Chem..

[21] Davies A., Kadurin I., Alvarez-Laviada A., Douglas L., Nieto-Rostro M., Bauer C.S., Pratt W.S., Dolphin A.C. (2010). The alpha2delta subunits of voltage-gated calcium channels form GPI-anchored proteins, a posttranslational modification essential for function. Proc. Natl. Acad. Sci. U. S. A..

[22] Tanabe T., Beam K.G., Adams B.A., Niidome T., Numa S. (1990). Regions of the skeletal muscle dihydropyridine receptor critical for excitation-contraction coupling. Nature.

[23] Nakai J., Tanabe T., Konno T., Adams B., Beam K.G. (1998). Localization in the II-III loop of the dihydropyridine receptor of a sequence critical for excitation-contraction coupling. J. Biol. Chem..

[24] Sheridan D.C., Cheng W., Ahern C.A., Mortenson L., Alsammarae D., Vallejo P., Coronado R. (2003). Truncation of the carboxyl terminus of the dihydropyridine receptor beta1a subunit promotes Ca2+ dependent excitation-contraction coupling in skeletal myotubes. Biophys. J..

[25] Eltit J.M., Franzini-Armstrong C., Perez C.F. (2014). Amino acid residues 489-503 of dihydropyridine receptor (dhpr) β1a subunit are critical for structural communication between the skeletal muscle dhpr complex and type 1 Ryanodine receptor. J. Biol. Chem..

[26] Sheridan D.C., Cheng W., Carbonneau L., Ahern C.A., Coronado R. (2004). Involvement of a heptad repeat in the carboxyl terminus of the dihydropyridine receptor beta1a subunit in the mechanism of excitation-contraction coupling in skeletal muscle. Biophys. J..

[27] Dayal A., Bhat V., Franzini-Armstrong C., Grabner M. (2013). Domain cooperativity in the β1a subunit is essential for dihydropyridine receptor voltage sensing in skeletal muscle. Proc. Natl. Acad. Sci. U. S. A..

[28] Yuchi Z., Van Petegem F. (2016). Ryanodine receptors under the magnifying lens: Insights and limitations of cryo-electron microscopy and X-ray crystallography studies. Cell Calcium.

[29] Abu-Omar N., Das J., Szeto V., Feng Z.-P. (2018). Neuronal ryanodine receptors in development and aging. Mol. Neurobiol..

[30] Zalk R., Clarke O.B., Des Georges A., Grassucci R.A., Reiken S., Mancia F., Hendrickson W.A., Frank J., Marks A.R. (2015). Structure of a mammalian ryanodine receptor. Nature.

[31] Efremov R.G., Leitner A., Aebersold R., Raunser S. (2015). Architecture and conformational switch mechanism of the ryanodine receptor. Nature.

[32] Yan Z., Bai X., Yan C., Wu J., Li Z., Xie T., Peng W., Yin C., Li X., Scheres S.H., Shi Y., Yan N. (2015). Structure of the rabbit ryanodine receptor RyR1 at near-atomic resolution. Nature.

[33] Franzini-Armstrong C. (1970). Studies of the triad: I. Structure of the junction in frog twitch fibers. J. Cell Biol..

[34] des Georges A., Clarke O.B., Zalk R., Yuan Q., Condon K.J., Grassucci R.A., Hendrickson W.A., Marks A.R., Frank J. (2016). Structural basis for gating and activation of RyR1. Cell.

[35] Van Petegem F. (2012). Ryanodine receptors: Structure and function. J. Biol. Chem..

[36] Woll K.A., Haji-Ghassemi O., Van Petegem F. (2021). Pathological conformations of disease mutant Ryanodine receptors revealed by cryo-EM. Nat. Commun..

[37] Iyer K.A., Hu Y., Nayak A.R., Kurebayashi N., Murayama T., Samso M. (2020). Structural mechanism of two gain-of-function cardiac and skeletal RyR mutations at an equivalent site by cryo-EM. Sci. Adv..

[38] Tae H.S., Norris N.C., Cui Y., Karunasekara Y., Board P.G., Dulhunty A.F., Casarotto M.G. (2009). Molecular recognition of the disordered dihydropyridine receptor II-III loop by a conserved spry domain of the type 1 ryanodine receptor. Clin. Exp. Pharmacol. Physiol..

[39] Lau K., Van Petegem F. (2014). Crystal structures of wild type and disease mutant forms of the ryanodine receptor SPRY2 domain. Nat. Commun..

[40] Block B.A., Imagawa T., Campbell K.P., Franzini-Armstrong C. (1988). Structural evidence for direct interaction between the moleuclar components of the T/SR junction in skeletal muscle. J. Cell Biol..

[41] Suzuki H., Kawai J., Taga C., Yaoi T., Hara A., Hirose K., Hayashizaki Y., Watanabe S. (1996). Stac, a novel neuron-specific protein with cysteine-rich and SH3 domains. Biochem. Biophys. Res. Commun..

[42] Horstick E.J., Linsley J.W., Dowling J.J., Hauser M.A., McDonald K.K., Ashley-Koch A., Saint-Amant L., Satish A., Cui W.W., Zhou W., Sprague S.M., Stamm D.S., Powell C.M., Speer M.C., Franzini-Armstrong C. (2013). Stac3 is a component of the excitation-contraction coupling machinery and mutated in native American myopathy. Nat. Commun..

[43] Nelson B.R., Wu F., Liu Y., Anderson D.M., Mcanally J., Lin W., Cannon S.C., Bassel-Duby R., Olson E.N. (2013). Skeletal muscle-specific T-tubule protein STAC3 mediates voltage-induced Ca 2+ release and contractility. Proc. Natl. Acad. Sci. U. S. A..

[44] Stamm D.S., Powell C.M., Stajich J.M., Zismann V.L., Stephan D.A., Chesnut B., Aylsworth A.S., Kahler S.G., Deak K.L., Gilbert J.R., Speer M.C. (2008). Novel congenital myopathy locus identified in native American Indians at 12q13.13-14.1. Neurology.

[45] Legha W., Gaillard S., Gascon E., Malapert P., Hocine M., Alonso S., Moqrich A. (2010). Stac1 and stac2 genes define discrete and distinct subsets of dorsal root ganglia neurons. Gene Expr. Patterns.

[46] Perni S., Lavorato M., Beam K.G., Ahern C.A., Nelson M.T. (2017). De novo reconstitution reveals the proteins required for skeletal muscle voltage-induced Ca2+ release. Proc. Natl. Acad. Sci. U. S. A..

[47] Takeshima H., Komazaki S., Nishi M., Iino M., Kangawa K. (2000). Junctophilins: A novel family of junctional membrane complex proteins. Mol. Cell.

[48] Polster A., Perni S., Bichraoui H., Beam K.G., Bean B.P., Snutch T.P. (2015). Stac adaptor proteins regulate trafficking and function of muscle and neuronal L-type Ca 2+ channels. Proc. Natl. Acad. Sci. U. S. A..

[49] Polster A., Nelson B.R., Olson E.N., Beam K.G., Brehm P., Grabner M. (2016). Stac3 has a direct role in skeletal muscle-type excitation–contraction coupling that is disrupted by a myopathy-causing mutation. Proc. Natl. Acad. Sci. U. S. A..

[50] Das J., Rahman G.M. (2014). C1 domains: Structure and ligand-binding properties. Chem. Rev..

[51] Chen D., Purohit A., Halilovic E., Doxsey S.J., Newton A.C. (2004). Centrosomal anchoring of protein kinase C betaII by pericentrin controls microtubule organization, spindle function, and cytokinesis. J. Biol. Chem..

[52] Anilkumar N., Parsons M., Monk R., Ng T., Adams J.C. (2003). Interaction of fascin and protein kinase Calpha: A novel intersection in cell adhesion and motility. EMBO J..

[53] Wang H., Kazanietz M.G. (2002). Chimaerins, novel non-protein kinase C phorbol ester receptors, associate with Tmp21-I (p23): Evidence for a novel anchoring mechanism involving the chimaerin C1 domain. J. Biol. Chem..

[54] Abe, T., Kurosaki, C., Yoshida, M., Hayashi, F., Hirota, H., and Yokoyama, S. Solution Structure of RSGI RUH-051, a C1 Domain of STAC3 From Human cDNA. 10.2210/pdb2db6/pdb

[55] Zhang G., Kazanietz M.G., Blumberg P.M., Hurley J.H. (1995). Crystal structure of the cys2 activator-binding domain of protein kinase C delta in complex with phorbol ester. Cell.

[56] Wong King Yuen S.M., Campiglio M., Tung C.-C., Flucher B.E., Van Petegem F. (2017). Structural insights into binding of STAC proteins to voltage-gated calcium channels. Proc. Natl. Acad. Sci. U. S. A..

[57] Rufenach B., Christy D., Flucher B.E., Bui J.M., Campiglio M., Gsponer J., Van Petegem F. (2020). Multiple sequence variants in STAC3 affect interactions with CaV1.1 and excitation-contraction coupling. Structure.

[58] Grabner M., Dirksen R.T., Suda N., Beam K.G. (1999). The II-III loop of the skeletal muscle dihydropyridine receptor is responsible for the bi-directional coupling with the Ryanodine receptor. J. Biol. Chem..

[59] Polster A., Nelson B.R., Papadopoulos S., Olson E.N., Beam K.G. (2018). Stac proteins associate with the critical domain for excitation-contraction coupling in the II-III loop of CaV1.1. J. Gen. Physiol..

[60] Linsley J.W., Hsu I.-U., Groom L., Yarotskyy V., Lavorato M., Horstick E.J., Linsley D., Wang W., Franzini-Armstrong C., Dirksen R.T., Kuwada J.Y. (2017). Congenital myopathy results from misregulation of a muscle Ca2+ channel by mutant Stac3. Proc. Natl. Acad. Sci. U. S. A..

[61] Zhao Y., Huang G., Wu J., Wu Q., Gao S., Yan Z., Lei J., Yan N. (2019). Molecular basis for ligand modulation of a mammalian voltage-gated Ca2+ channel. Cell.

[62] Johnson B.D., Brousal J.P., Peterson B.Z., Gallombardo P.A., Hockerman G.H., Lai Y., Scheuer T., Catterall W.A. (1997). Modulation of the cloned skeletal muscle L-type Ca2+ channel by anchored cAMP-dependent protein kinase. J. Neurosci..

[63] Zong X., Hofmann F. (1996). Ca2+-dependent inactivation of the class C L-type Ca2+ channel is a property of the a1 subunit. FEBS Lett..

[64] Yasuda T., Chen L., Barr W., McRory J.E., Lewis R.J., Adams D.J., Zamponi G.W. (2004). Auxiliary subunit regulation of high-voltage activated calcium channels expressed in mammalian cells. Eur. J. Neurosci..

[65] Niu J., Yang W., Yue D.T., Inoue T., Ben Johny M. (2018). Duplex signaling by CaM and Stac3 enhances Cav1.1 function and provides insights into congenital myopathy. J. Gen. Physiol..

[66] Linsley J.W., Hsu I.U., Wang W., Kuwada J.Y. (2017). Transport of the alpha subunit of the voltage gated L-type calcium channel through the sarcoplasmic reticulum occurs prior to localization to triads and requires the beta subunit but not Stac3 in skeletal muscles. Traffic.

[67] Rzhepetskyy Y., Lazniewska J., Proft J., Campiglio M., Flucher B.E., Weiss N. (2016). A Cav3.2/Stac1 molecular complex controls T-type channel expression at the plasma membrane. Channels.

[68] Wu F., Quinonez M., DiFranco M., Cannon S.C. (2018). Stac3 enhances expression of human CaV1.1 in Xenopus oocytes and reveals gating pore currents in HypoPP mutant channels. J. Gen. Physiol..

[69] Campiglio M., Flucher B.E. (2017). STAC3 stably interacts through its C1 domain with Ca V 1.1 in skeletal muscle triads. Sci. Rep..

[70] Campiglio M., Flucher B.E. (2018). STAC3 incorporation into skeletal muscle triads occurs independent of the dihydropyridine receptor. J. Cell. Physiol..

[71] Peterson B.Z., DeMaria C.D., Yue D.T. (1999). Calmodulin is the Ca2+ sensor for Ca2+-dependent inactivation of L-type calcium channels. Neuron.

[72] Qin N., Olcese R., Bransby M., Lin T., Birnbaumer L. (1999). Ca2+-induced inhibition of the cardiac Ca2+ channel depends on calmodulin. Proc. Natl. Acad. Sci. U. S. A..

[73] Zühlke R.D., Pitt G.S., Deisseroth K., Tsien R.W., Reuter H. (1999). Calmodulin supports both inactivation and facilitation of L-type calcium channels. Nature.

[74] Dick I.E., Tadross M.R., Liang H., Tay L.H., Yang W., Yue D.T. (2008). A modular switch for spatial Ca2+ selectivity in the calmodulin regulation of CaV channels. Nature.

[75] Polster A., Perni S., Bichraoui H., Beam K.G. (2015). Stac adaptor proteins regulate trafficking and function of muscle and neuronal L-type Ca2+ channels. Proc. Natl. Acad. Sci. U. S. A..

[76] Polster A., Dittmer P.J., Perni S., Bichraoui H., Sather W.A., Beam K.G. (2018). Stac proteins suppress Ca2+-dependent inactivation of neuronal L-type Ca2+ channels. J. Neurosci..

[77] Niu J., Dick I.E., Yang W., Bamgboye M.A., Yue D.T., Tomaselli G., Inoue T., Ben-Johny M. (2018). Allosteric regulators selectively prevent Ca 2+ -feedback of Ca v and Na v channels. Elife.

[78] Campiglio M., Costé de Bagneaux P., Ortner N.J., Tuluc P., Van Petegem F., Flucher B.E. (2018). STAC proteins associate to the IQ domain of CaV1.2 and inhibit calcium-dependent inactivation. Proc. Natl. Acad. Sci. U. S. A..

[79] Yoder J.B., Ben-Johny M., Farinelli F., Srinivasan L., Shoemaker S.R., Tomaselli G.F., Gabelli S.B., Amzel L.M. (2019). Ca2+-dependent regulation of sodium channels NaV1.4 and NaV1.5 is controlled by the post-IQ motif. Nat. Commun..

[80] Gabelli S.B., Boto A., Kuhns V.H., Bianchet M.A., Farinelli F., Aripirala S., Yoder J., Jakoncic J., Tomaselli G.F., Amzel L.M. (2014). Regulation of the NaV1.5 cytoplasmic domain by calmodulin. Nat. Commun..

[81] Gardill B.R., Rivera-Acevedo R.E., Tung C.-C., Van Petegem F. (2019). Crystal structures of Ca(2+)-calmodulin bound to Na(V) C-terminal regions suggest role for EF-hand domain in binding and inactivation. Proc. Natl. Acad. Sci. U. S. A..

[82] Wang C., Chung B.C., Yan H., Wang H.-G., Lee S.-Y., Pitt G.S. (2014). Structural analyses of Ca^2+^/CaM interaction with NaV channel C-termini reveal mechanisms of calcium-dependent regulation. Nat. Commun..

[83] Wang C., Chung B.C., Yan H., Lee S.-Y., Pitt G.S. (2012). Crystal structure of the ternary complex of a NaV C-terminal domain, a fibroblast growth factor homologous factor, and calmodulin. Structure.

[84] Flucher B.E., Tuluc P. (2017). How and why are calcium currents curtailed in the skeletal muscle voltage-gated calcium channels?. J. Physiol..

[85] Fisher T.E., Bourque C.W. (1995). Voltage-gated calcium currents in the magnocellular neurosecretory cells of the rat supraoptic nucleus. J. Physiol..

[86] Avery R.B., Johnston D. (1996). Multiple channel types contribute to the low-voltage-activated calcium current in hippocampal CA3 pyramidal neurons. J. Neurosci..

[87] Beck H., Steffens R., Heinemann U., Elger C.E. (1997). Properties of voltage-activated Ca2+ currents in acutely isolated human hippocampal granule cells. J. Neurophysiol..

[88] Cong X., Doering J., Mazala D.A.G., Chin E.R., Grange R.W., Jiang H. (2016). The SH3 and cysteine-rich domain 3 (Stac3) gene is important to growth, fiber composition, and calcium release from the sarcoplasmic reticulum in postnatal skeletal muscle. Skelet. Muscle.

[89] Reinholt B.M., Ge X., Cong X., Gerrard D.E., Jiang H. (2013). Stac3 is a novel regulator of skeletal muscle development in mice. PLoS One.

[90] Zierath J.R., Hawley J.A. (2004). Skeletal muscle fiber type: Influence on contractile and metabolic properties. PLoS Biol..

[91] Ge X., Zhang Y., Park S., Cong X., Gerrard D.E., Jiang H. (2014). Stac3 inhibits myoblast differentiation into myotubes. PLoS One.

[92] Zhang Y., Cong X., Wang A., Jiang H. (2014). Identification of the STAC3 gene as a skeletal muscle-specifically expressed gene and a novel regulator of satellite cell differentiation in cattle. J. Anim. Sci..

[93] Bower N.I., Garcia De La Serrana D., Cole N.J., Hollway G.E., Lee H.T., Assinder S., Johnston I.A. (2012). Stac3 is required for myotube formation and myogenic differentiation in vertebrate skeletal muscle. J. Biol. Chem..

[94] Telegrafi A., Webb B.D., Robbins S.M., Speck-Martins C.E., FitzPatrick D., Fleming L., Redett R., Dufke A., Houge G., van Harssel J.J.T., Verloes A., Robles A., Manoli I., Engle E.C., Jabs E.W. (2017). Identification of STAC3 variants in non-native American families with overlapping features of Carey–Fineman–Ziter syndrome and Moebius syndrome. Am. J. Med. Genet. A.

[95] Grzybowski M., Schänzer A., Pepler A., Heller C., Neubauer B.A., Hahn A. (2017). Novel STAC3 mutations in the first non-Amerindian patient with native American myopathy. Neuropediatrics.

[96] Zaharieva I., Sarkozy A., Munot P., Manzur A., O’Grady G., Rendu J., Malfatti E., Amthor H., Servais L., Urtizberea J.A., Neto O.A., Zanoteli E., Donkervoort S., Taylor J., Dixon J. (2018). STAC3 variants cause a congenital myopathy with distinctive dysmorphic features and malignant hyperthermia susceptibility. Hum. Mutat..

[97] Waldrop M.A., Boue D.R., Sites E., Flanigan K.M., Shell R. (2017). Clinicopathologic conference: A newborn with hypotonia, cleft palate, micrognathia, and bilateral club feet. Pediatr. Neurol..

[98] Stamm D.S., Aylsworth A.S., Stajich J.M., Kahler S.G., Thorne L.B., Speer M.C., Powell C.M. (2008). Native American myopathy: Congenital myopathy with cleft palate, skeletal anomalies, and susceptibility to malignant hyperthermia. Am. J. Med. Genet. A.

[99] Webb B.D., Manoli I., Jabs E.W., Adam M.P., Ardinger H.H., Pagon R.A. (1993-2021). STAC3 Disorder. GeneReviews® [Internet].

[100] Riazi S., Kraeva N., Hopkins P.M. (2018). Malignant hyperthermia in the post-genomics era: New perspectives on an old concept. Anesthesiology.

[101] Bailey A.G., Bloch E.C. (1987). Malignant hyperthermia in a three-month-old American Indian infant. Anesth. Analg..

[102] Fernandes C.R., Pinto Filho W.A., Cezar L.C., Gomes J.M.A., da Cunha G.K.F. (2013). Fatal recrudescence of malignant hyperthermia in an infant with Moebius syndrome. Braz. J. Anesthesiol..

[103] Pancaroglu R., Van Petegem F. (2018). Calcium channelopathies: Structural insights into disorders of the muscle excitation–contraction complex. Annu. Rev. Genet..

[104] Mellor G.J., Panwar P., Lee A.K., Steinberg C., Hathaway J.A., Bartels K., Christian S., Balaji S., Roberts J.D., Simpson C.S., Boczek N.J., Tester D.J., Radbill A.E., Mok N.S., Hamilton R.M. (2019). Type 8 long QT syndrome: Pathogenic variants in CACNA1C-encoded Cav1.2 cluster in STAC protein binding site. Europace.

[105] Fromer M., Pocklington A.J., Kavanagh D.H., Williams H.J., Dwyer S., Gormley P., Georgieva L., Rees E., Palta P., Ruderfer D.M., Carrera N., Humphreys I., Johnson J.S., Roussos P., Barker D.D. (2014). De novo mutations in schizophrenia implicate synaptic networks. Nature.

[106] Ambalavanan A., Girard S.L., Ahn K., Zhou S., Dionne-Laporte A., Spiegelman D., Bourassa C.V., Gauthier J., Hamdan F.F., Xiong L., Dion P.A., Joober R., Rapoport J., Rouleau G.A. (2016). De novo variants in sporadic cases of childhood onset schizophrenia. Eur. J. Hum. Genet..

[107] Satoh J.I., Nanri Y., Yamamura T. (2006). Rapid identification of 14-3-3-binding proteins by protein microarray analysis. J. Neurosci. Methods.

[108] Hardy K., Mansfield L., Mackay A., Benvenuti S., Ismail S., Arora P., O’Hare M.J., Jat P.S. (2005). Transcriptional networks and cellular senescence in human mammary fibroblasts. Mol. Biol. Cell.

[109] Jeong E., Choi H.K., Park J.H., Lee S.Y. (2018). STAC2 negatively regulates osteoclast formation by targeting the RANK signaling complex. Cell Death Differ..

[110] Bao Y., Wang L., Shi L., Yun F., Liu X., Chen Y., Chen C., Ren Y., Jia Y. (2019). Transcriptome profiling revealed multiple genes and ECM-receptor interaction pathways that may be associated with breast cancer. Cell. Mol. Biol. Lett..

[111] Yang H., Zhou L., Chen J., Su J., Shen W., Liu B., Zhou J., Yu S., Qian J. (2019). A four-gene signature for prognosis in breast cancer patients with hypermethylated IL15RA. Oncol. Lett..

[112] Bi X., Liu J., Xu S., Wang Y., Wu X. (2021). Testicular STAC3 regulates Leydig cell steroidogenesis through potentiating mitochondrial membrane potential and StAR processing. Cell Tissue Res..

[113] Dickinson M.E., Flenniken A.M., Ji X., Teboul L., Wong M.D., White J.K., Meehan T.F., Weninger W.J., Westerberg H., Adissu H., Baker C.N., Bower L., Brown J.M., Caddle L.B., Chiani F. (2016). High-throughput discovery of novel developmental phenotypes. Nature.

[114] Hsu I., Linsley J.W., Zhang X., Varineau J.E., Berkhoudt D.A., Reid L.E., Lum M., Orzel A.M., Leflein A., Xu H., Collins C.A., Hume R.I., Levitan E.S., Kuwada J.Y. (2020). Stac protein regulates release of neuropeptides. Proc. Natl. Acad. Sci. U. S. A..

[115] Hsu I.U., Linsley J.W., Varineau J.E., Shafer O.T., Kuwada J.Y. (2018). Dstac is required for normal circadian activity rhythms in Drosophila. Chronobiol. Int..

[116] Huttlin E.L., Bruckner R.J., Paulo J.A., Cannon J.R., Ting L., Baltier K., Colby G., Gebreab F., Gygi M.P., Parzen H., Szpyt J., Tam S., Zarraga G., Pontano-Vaites L., Swarup S. (2017). Architecture of the human interactome defines protein communities and disease networks. Nature.

[117] Huttlin E.L., Ting L., Bruckner R.J., Gebreab F., Gygi M.P., Szpyt J., Tam S., Zarraga G., Colby G., Baltier K., Dong R., Guarani V., Vaites L.P., Ordureau A., Rad R. (2015). The BioPlex network: A systematic exploration of the human interactome. Cell.

[118] Rolland T., Taşan M., Charloteaux B., Pevzner S.J., Zhong Q., Sahni N., Yi S., Lemmens I., Fontanillo C., Mosca R., Kamburov A., Ghiassian S.D., Yang X., Ghamsari L., Balcha D. (2014). A proteome-scale map of the human interactome network. Cell.

[119] Luck K., Kim D.-K., Lambourne L., Spirohn K., Begg B.E., Bian W., Brignall R., Cafarelli T., Campos-Laborie F.J., Charloteaux B., Choi D., Coté A.G., Daley M., Deimling S., Desbuleux A. (2020). A reference map of the human binary protein interactome. Nature.

[120] Thalappilly S., Suliman M., Gayet O., Soubeyran P., Hermant A., Lecine P., Iovanna J.L., Dusetti N.J. (2008). Identification of multi-SH3 domain-containing protein interactome in pancreatic cancer: A yeast two-hybrid approach. Proteomics.

[121] Troiani S., Lupi R., Perego R., Depaolini S.R., Thieffine S., Bosotti R., Rusconi L. (2011). Identification of candidate substrates for poly(ADP-ribose) polymerase-2 (PARP2) in the absence of DNA damage using high-density protein microarrays. FEBS J..

[122] Rual J.-F., Venkatesan K., Hao T., Hirozane-Kishikawa T., Dricot A., Li N., Berriz G.F., Gibbons F.D., Dreze M., Ayivi-Guedehoussou N., Klitgord N., Simon C., Boxem M., Milstein S., Rosenberg J. (2005). Towards a proteome-scale map of the human protein-protein interaction network. Nature.

[123] Chen S., Fragoza R., Klei L., Liu Y., Wang J., Roeder K., Devlin B., Yu H. (2018). An interactome perturbation framework prioritizes damaging missense mutations for developmental disorders. Nat. Genet..

[124] Yu H., Tardivo L., Tam S., Weiner E., Gebreab F., Fan C., Svrzikapa N., Hirozane-Kishikawa T., Rietman E., Yang X., Sahalie J., Salehi-Ashtiani K., Hao T., Cusick M.E., Hill D.E. (2011). Next-generation sequencing to generate interactome datasets. Nat. Methods.

